# Antimicrobial Management of Disseminated Strongyloidiasis in a COVID-19 Patient

**DOI:** 10.4269/ajtmh.20-1059b

**Published:** 2020-11

**Authors:** Audun J. Lier, Matthew W. Davis, Jeffrey E. Topal

**Affiliations:** Section of Infectious Diseases; Department of Internal Medicine; Yale University School of Medicine; New Haven, Connecticut; E-mail: audun.lier@yale.edu; Department of Pharmacy Services; Yale New Haven Hospital; New Haven, Connecticut; E-mail: matthew.davis2@ynhh.org; Section of Infectious Diseases; Department of Internal Medicine; Yale University School of Medicine; New Haven, Connecticut; Department of Pharmacy Services; Yale New Haven Hospital; New Haven, Connecticut; E-mail: jeffrey.topal@ynhh.org; Department of Pharmacy Services and the Section of Infectious Diseases; Yale University School of Medicine; New Haven, Connecticut

Dear Sir,

The authors raise the issue of early discontinuation of antibiotic therapy in a patient with presumed strongyloidiasis. In our patient’s case, antibiotic therapy had been discontinued on hospital day 19 following initiation of ivermectin therapy for disseminated strongyloides infection.^1^ The patient had received broad-spectrum antibiotic therapy since day 12, and his blood cultures, which previously grew Gram-positive and Gram-negative bacteria, had cleared by day 14.^2^ The decision to discontinue antibiotic therapy on this date was based on clinical improvement leading to extubation, a decrease in white blood cell count, a procalcitonin level in the normal range, and the diagnosis of a nonbacterial infection. The presence of persistent fevers and development of altered mental status after the initiation of ivermectin led to the consideration of bacterial meningitis, which is a well-known complication of disseminated strongyloidiasis.^3^ Consequently, he was restarted on antibiotic therapy, and a lumbar puncture was recommended. Unfortunately, a lumbar puncture could not be performed, and he was treated empirically for bacterial meningitis.

The World Gastroenterology Organization global guidelines on management of strongyloidiasis state “Hyperinfection carries a high risk of Gram-negative septicemia, so broad-spectrum antibiotics are usually given, especially to prevent bacterial meningitis.”^4^ However, we are unaware of published data supporting using antibiotics to prevent meningitis in this context, and no reference is provided in the guidelines. In our case, it is unclear if the patient had bacterial meningitis because of the inability to obtain a lumbar puncture. The patient’s clinical status improved not only with antibiotics but also with the addition of albendazole, which makes it difficult to discern the cause of the clinical decompensation. Clinical decisions must be individualized, but we agree that it may have been reasonable to continue antibiotic therapy after starting ivermectin.
